# Flap-Free Tendon Coverage Using Autologous Fat Grafts Enhanced with Platelet-Rich Plasma and Growth Factors at a Secondary Level Hospital: A Case Report

**DOI:** 10.3390/jcm13185640

**Published:** 2024-09-23

**Authors:** Guadalupe Santamaría Salvador, Esteban Acosta Muñoz, Juan Samaniego Rojas, Charles Hidalgo Quishpe, Juan S. Izquierdo-Condoy, Jorge Vasconez-Gonzalez, Esteban Ortiz-Prado

**Affiliations:** 1 Plastic and Reconstructive Department, Hospital Vozandes, Quito 170521, Ecuador; 2 One Health Research Group, Faculty of Health Science, Universidad de Las Americas, Quito 170513, Ecuador

**Keywords:** autologous fat graft, tendon exposure, platelet-rich plasma, wound healing, regenerative medicine

## Abstract

Background: Autologous fat grafting, enriched with platelet-rich plasma (PRP), has been established as an effective and affordable treatment for various types of wound healing. However, its efficacy in managing wounds with tendon exposure has not been thoroughly investigated. Methods: We report the case of a 40-year-old male who sustained a severe friction burn on his hand and forearm from a car accident, resulting in significant tissue loss and exposed extensor tendons. Results: Traditional wound treatment strategies were not implemented due to specific patient circumstances. After initial surgical management failed to prevent necrosis and maintain coverage of the exposed tendons, the patient underwent a novel treatment involving autologous fat grafting combined with PRP and growth factors. The procedure was repeated twice within a month to promote granular tissue formation over that area and facilitate subsequent coverage with an epidermoreticular graft. By day 21 post-initial graft, the exposed tendons were 98% covered with granular tissue. Complete wound coverage was achieved by day 60, and by day 130 the patient had regained 90% functionality of the affected limbs. Conclusions: This case illustrates the potential of autologous fat grafting combined with PRP and growth factors as a viable, flap-free alternative for covering tendon exposures. This approach not only enhances wound healing but also supports functional recovery, underscoring the need for further research into its broader applicative potentials.

## 1. Introduction

Fat grafting, commonly referred to as “lipofilling,” is a sophisticated surgical procedure that involves the transfer of autologous adipose tissue from one bodily region to another [1]. This technique, facilitated through minimally invasive liposuction, not only proves to be well-tolerated by patients but also stands as a safe and effective approach for various therapeutic and aesthetic applications [2]. Recent advancements have highlighted adipose tissue as a rich reservoir of adult stem cells—more concentrated than any other tissue type—which exhibit potential for cellular differentiation across various tissue types, including adipose, bone, muscle, cartilage, nervous, and vascular tissues [3]. The stromal vascular fraction (SVF) within adipose tissue is particularly valued for its reparative and regenerative capabilities [4,5].

With the rapid development of transportation and construction industries, skin and subcutaneous soft tissue defects have become increasingly common. Such injuries often result in the exposure of critical underlying structures such as tendons and bones, posing significant clinical challenges. The treatment of large and refractory wounds requires advanced soft tissue management techniques to avoid complications like infection, scar contracture, and potentially, disability [6,7,8]. These scenarios typically necessitate interventions that range from simple second-intention healing to more complex procedures like skin grafting with or without dermal substitutes, or local to distant flaps [6,7,8,9].

Conventional treatments often involve debridement followed by the transplantation of skin flaps [10]. However, this can result in significant donor site morbidity and may not be suitable due to the poor vascularity, reduced tissular partial pressure of oxygen (PtO2) at the defect sites, or reduced local growth factor activity [11,12]. Platelet-rich plasma (PRP), a regenerative biomaterial, has gained attention for its high concentrations of growth factors such as platelet-derived growth factor (PDGF), transforming growth factor-beta (TGF-β), and vascular endothelial growth factor (VEGF), which promote angiogenesis and tissue repair at the defect sites [13,14]. When combined with autologous fat grafting, PRP not only enhances the survival of the grafted tissue but also supports angiogenesis and cellular differentiation, making this combination a potent option for managing complex wounds [15,16].

This study explores the therapeutic potentials of autologous fat grafting combined with PRP in the treatment of a severe case involving tendon exposure following a traffic accident. The case underscores the challenges of healing poorly vascularized defects and the innovative application of combined regenerative therapies to facilitate functional recovery and aesthetic improvement.

## 2. Case Report

A 40-year-old male with no significant personal or family medical history was presented to the emergency department following a traffic accident that resulted in trauma to his left hand and forearm. The injuries occurred when his left upper limb was forcibly displaced and came into contact with the pavement.

**Figure 1:**
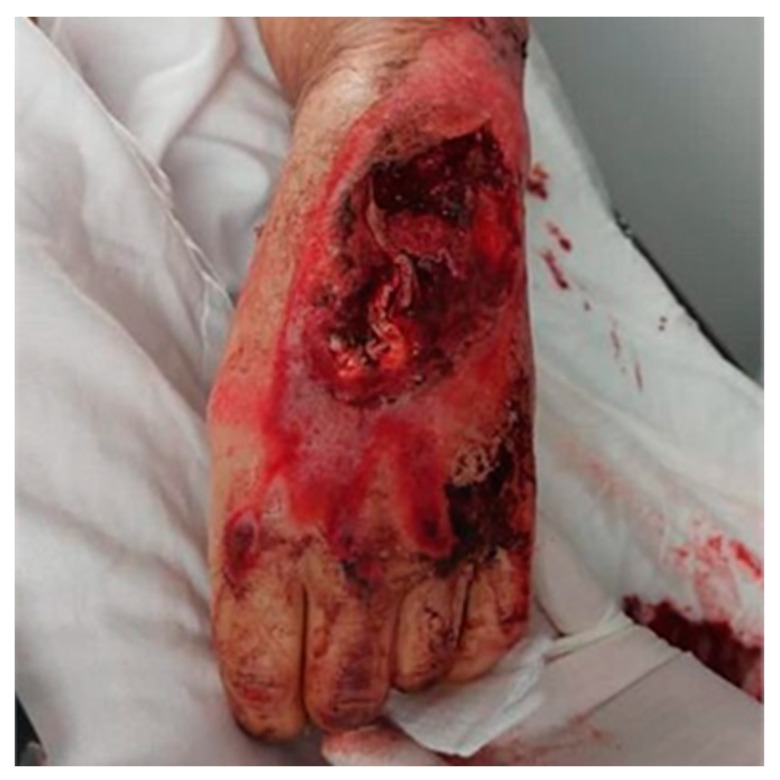
Initial clinical presentation of the left hand and forearm injuries. This image displays extensive trauma to the patient’s left hand and forearm resulting from a traffic accident. Visible are the friction burn and the subsequent loss of skin measuring 8 cm × 7 cm on the dorsum of the hand. This figure also shows a detachment of the extensor aponeurotic system from the second to fifth tendons, along with a 5 cm × 2.5 cm wound located on the ulnar border of the forearm’s middle third. Notable is the avulsion of the cortex of the distal epiphysis of the radius. The exposed tendons and surrounding muscle tissues are clearly delineated, highlighting the complex nature of the injuries sustained.

Upon stabilization in the emergency department, the patient was evaluated for fractures, which were ruled out. The plastic surgery team conducted a thorough examination, revealing a significant friction burn on the left hand and forearm, with a tissue loss area measuring approximately 8 cm × 7 cm on the dorsum of the left hand. Additionally, there was a detachment of the extensor aponeurotic system involving the second to fifth tendons. A separate wound, 5 cm × 2.5 cm, was found on the ulnar border of the left forearm. An avulsion injury to the cortex of the distal epiphysis of the radius was also noted. No signs of compartment syndrome were present (Figure 1).

### 2.1. Initial Management and Surgical Intervention

Upon admission, the patient was promptly started on antibiotic therapy with a combination of Ampicillin and Sulbactam at a dosage of 3 g to prevent infection. Within six hours of the accident, the patient underwent surgery, where extensive debridement was performed to clean the wound. Tenorrhaphy of the second to fifth extensor tendons was completed using the Bunnell technique. The wound on the dorsum of the hand was primarily closed with the remaining perilesional skin. The hand was immobilized in a 90-degree hyperextension using an anterior splint, which was to remain in place for 15 days. The wound edges on the forearm were approximated and closed by secondary intention to prevent compartment syndrome.

### 2.2. Postoperative Care

Fifteen minutes post-surgery, the splint was temporarily removed to check for any signs of circulatory or nerve compression before being reapplied. Four days post-surgery, irrigation was performed with 500 cc of 0.9% saline solution, and the hydrocolloid bandage (Duoderm) was changed. This process was repeated every four days. Wound cultures were performed every eight days until the first fat graft, all of which were negative for bacterial superinfection. Physical therapy began on the fifth day, following an early mobilization protocol to prevent damage to other structures due to the hyperextension position of the hand. The hyperextension splint was changed to a dynamic splint in the first week. Physical therapy included four phases: immobilization and early controlled mobilization (0–3 weeks), active-assisted mobilization (4–6 weeks), full active mobilization (7–12 weeks), and strengthening with a return to full function (13 weeks onwards).

### 2.3. Complication Management

**Figure 2:**
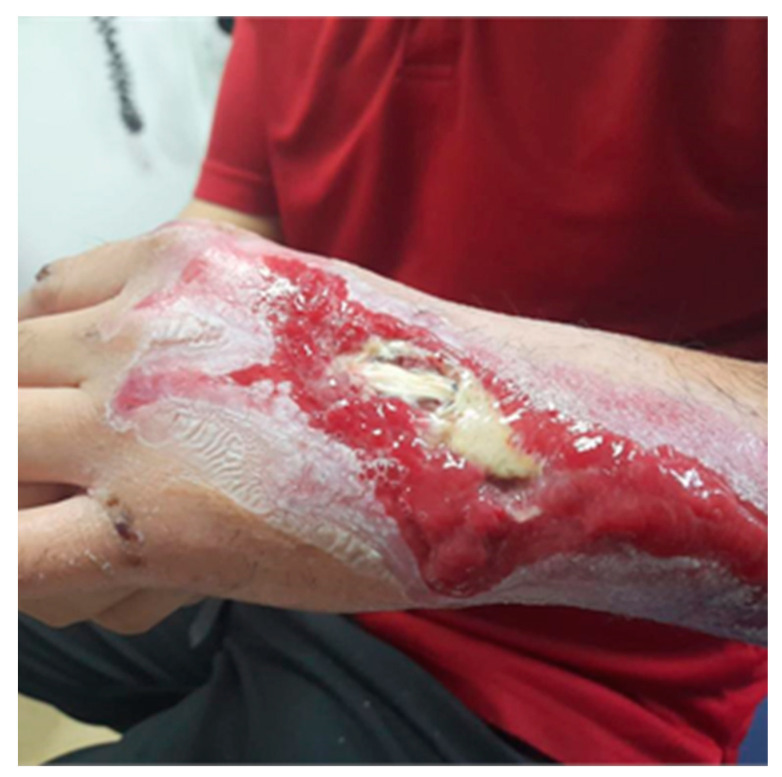
Initial postoperative necrosis with exposed tendons and granulation tissue.

On the twenty-ninth day following surgery, a 4 × 4 cm area of necrosis with tendon exposure was identified on the dorsum of the affected hand, involving the second to fourth extensor tendons, accompanied by perilesional granulation tissue (Figure 2). The patient declined further surgical interventions such as local or free microsurgical skin flap. Consequently, it was decided to treat the exposed tendons using an autologous fat graft augmented with PRP and growth factors, aiming to leverage their regenerative capabilities.

### 2.4. Autologous Fat Grafting Technique and Platelet-Rich Plasma Preparation

The required instruments for the fat graft pooling technique included saline solution (0.9%), epinephrine (dilution 1:1,000,000), bicarbonate, 2% lidocaine, a 3 mm liposuction cannula for fat grafting, a 10 cc syringe (vacuum syringe), and Vaseline gauze.

Autologous fat was harvested from the abdominal panniculus, specifically the periumbilical area, during both the first and second sessions. The donor site was infiltrated using a tumescent technique with a solution of 0.9% saline, 1:1,000,000 epinephrine, 10 mEq of bicarbonate, and 10 cc of 2% lidocaine without epinephrine. A 3 mm liposuction cannula was then used to harvest approximately 10 cc of fat tissue per session. The harvested fat was processed via decantation in the same collection syringe for 3 min. The lower portion was then removed, isolating the fat. The isolated fat was then mixed with 5 cc of PRP activated with 1 cc of thrombin to release growth factors. The procedure described corresponded to the preparation of platelet-rich plasma (PRP), which was activated with thrombin at the time of use to promote the release of growth factors.

Blood Collection: Venipuncture was performed to collect between 15 and 20 cc of the patient’s blood into 3.5 mL tubes containing sodium citrate as an anticoagulant (blue cap). The volume of blood collected may vary depending on the patient’s hematocrit. In this case, the patient’s hematocrit was 45.5%, which is within the typical range of 40–50% for a healthy young male.

Centrifugation: The tubes were centrifuged immediately after collection at high speeds (6000–8000 rpm) for 15 min at room temperature. Following centrifugation, the blood was fractionated and the plasma was extracted while avoiding leukocytes.

Platelet Count: While the hospital protocol does not include an individualized platelet count for the PRP, it is estimated, based on institutional experience, that the platelet concentration in PRP is approximately five times higher than in peripheral blood. In this case, the patient had a peripheral blood platelet count of 206,000 platelets per microliter.

PRP Activation: To activate the platelets in PRP and facilitate the release of growth factors such as platelet-derived growth factor (PDGF), transforming growth factor-beta (TGF-β), and vascular endothelial growth factor (VEGF), a secondary protocol, termed “Obtaining Autologous Thrombin,” was followed.

This procedure was performed at the time of PRP use. Blood was collected from the patient into tubes without additives (red cap) with volumes ranging from 15 to 20 cc. The tube was centrifuged at a low speed (4000 rpm) for 10 min at room temperature. Subsequently, a fibrin clot was formed, and thrombin was obtained from the residual serum. For activation, the protocol specifies using between 0.5 cc and 1 cc of thrombin. In this case, 1 cc of thrombin was utilized, and it is important to note that calcium chloride was not employed in this procedure.

**Figure 3:**
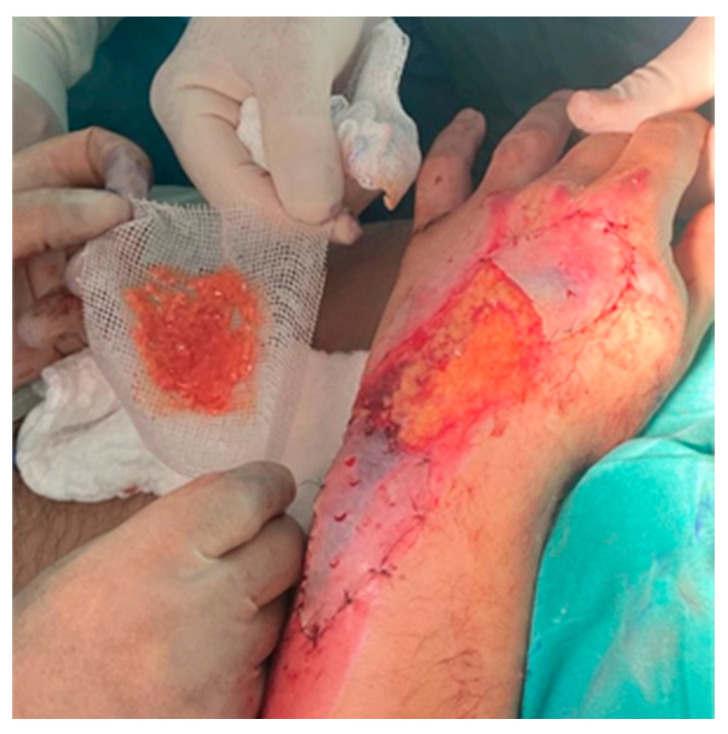
Application of autologous fat graft in the middle third (4 × 4 cm area of necrosis with tendon exposure on the dorsum of the affected hand, involving the second to fourth extensor tendons) and partial-thickness ER grafts in the lateral thirds (adjacent to the necrotic areas).

The mixture of autologous fat and PRP was applied to Vaseline gauze, which was then placed over the exposed tendons on the dorsum of the hand, specifically covering the 4 × 4 cm area of necrosis with tendon exposure. This was further covered with saline-soaked gauze and then with a gauze bandage. Concurrently, partial-thickness epidermoreticular (ER) grafts were placed adjacent to the necrotic area over the granulating tissue to promote skin regeneration on the dorsum of the affected hand and over the wound located in the middle third of the left forearm along the ulnar border, which was initially managed by secondary intention to prevent compartment syndrome (Figure 3).

### 2.5. Follow-Up and Outcome

Three days after the initial fat graft application, 50% graft vitality was noted, prompting a second application of adipose tissue combined with PRP and growth factors, particularly targeting the necrotic areas. By the fourth day following this second intervention, graft vitality had improved to 90%. Hydrocolloid dressings were maintained until approximately 98% tissue degranulation was achieved, followed by a final ER grafting. Sixty days post-second grafting, complete wound coverage was observed.

**Figure 4:**
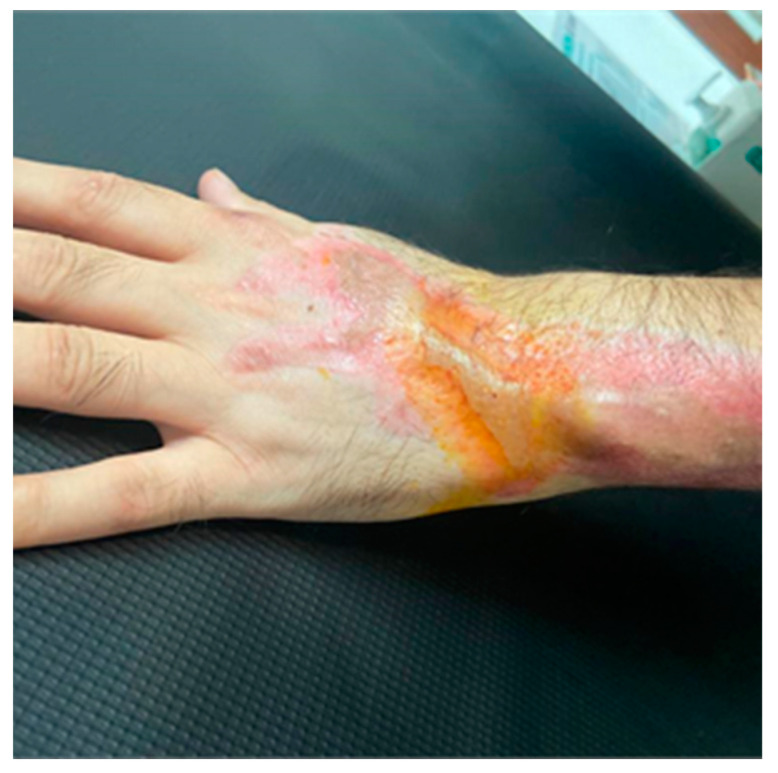
Final result of healing showing complete wound coverage accompanied by functional recovery.

At 130 days after the first surgical intervention, although specific range-of-motion measurements for each tendon were not performed, functional recovery was deemed satisfactory based on the patient’s ability to perform daily activities without significant restrictions or pain. The patient denied experiencing weakness, pain, paresthesias, or sensory loss in the back of the forearm and hand, except in the area where the first and second autologous fat grafts were performed, which exhibited some sensory loss. No muscle atrophy was observed in the muscles innervated by the radial and posterior interosseous nerves. The patient was able to perform full functional flexion and extension movements in the second to fifth fingers (Supplementary Material S1). Elbow extension against resistance, wrist extension, and thumb extension against resistance were normal, with no sign of dropped wrist evident. Sensitivity was preserved in the back of the arm, forearm, and hand (first interdigital space), except in the aforementioned area. The patient’s self-assessment indicated a 90% recovery of functionality (Figure 4). The DASH questionnaire in the disability scoring module yielded a score of 12, indicating low disability (Supplementary Material S2). Although the traumatologists recommended surgical correction for the avulsion injury to the cortex of the distal epiphysis of the radius, the patient declined, given that his hand functionality in daily activities was not affected, and the integrity of the achieved skin coverage was a priority.

## 3. Discussion

This report details the outcomes of using autologous fat grafting augmented with PRP and growth factors to manage a wound with exposed tendons resulting from a traffic accident, a scenario that poses considerable healing challenges. Traditional approaches, such as local or remote skin flaps, are often preferred for primary wound repair, especially in cases involving poorly vascularized defects like exposed bones or tendons [15]. However, due to the unique characteristics of the lesion and the patient’s preference against conventional flap techniques, these methods were not suitable.

Instead, a novel approach involving autologous fat grafting combined with PRP and growth factors was employed, offering a promising alternative that facilitated significant functional recovery. The primary advantage of this technique lies in the rich presence of connective tissue cells within the stromal vascular fraction of adipose tissue, which includes a diverse cell population such as preadipocytes, endothelial cells, monocytes, macrophages, granulocytes, lymphocytes, and notably, adipose-derived stem cells (ADSCs). These cells are integral to promoting wound healing through mechanisms like enhanced cellular proliferation, differentiation, reduced inflammation, and improved vascularization [16,17,18].

The procedure’s low invasiveness and ease of access further reduce the risks associated with more invasive sources like bone marrow [11,12]. Additionally, the role of PRP in accelerating the healing process of traumatic wounds and ulcers is well-established, with benefits stemming from the high concentration of platelet-derived growth factors enhancing tissue repair and angiogenesis [19,20]

Despite these advantages, the technique is not devoid of potential complications, which can include bleeding, infection, and, in rare cases, graft hypertrophy from excessive collagen deposition [21]. Extensive testing in both animal and human clinical studies has validated the efficacy of autologous fat grafting combined with PRP across various wound types and anatomical locations. Yet, the literature remains scant on its application in wounds with significant tendon exposure, as demonstrated in this case [22,23,24].

This gap is notable, although the technique has been explored for other applications such as filling depressed scars and covering bone exposures. For instance, Kao et al. reported that fat grafts, used alongside negative pressure therapy, facilitated mesenchymal healing in a murine model, creating enough granulation tissue for effective bone coverage suitable for subsequent skin grafting [25]. Similarly, Rangaswamy M. described successful outcomes in a series involving patients with bone exposure using the same intervention strategy [26].

These findings from animal models underscore the potential of autologous fat graft and PRP therapy in complex clinical scenarios involving exposed tissues that carry functional risks, such as bones and tendons. The absence of extensive clinical reports using this regenerative therapy as a tissue bridge in deep wounds highlights the innovative aspect of our approach and underscores the need for further research. This would not only validate the efficacy and safety of this technique but also refine its application protocols, maximizing therapeutic outcomes in clinical practice.

From a public health perspective, traditional wound care methods, such as prolonged use of colloid patches, hydrocolloids, alginate dressings, and platelet gels, often require extensive resource utilization over long treatment periods [27,28]. These conventional treatments can become economically burdensome due to the high costs of long-term care and frequent dressing changes required to manage chronic wounds. Furthermore, they may lead to delayed reintegration into the workforce due to prolonged recovery times.

In contrast, the use of autologous fat grafts combined with PRP offers a more efficient solution via potentially reducing the healing time and improving the functional recovery of patients. This not only minimizes direct medical costs through curtailing the need for repetitive and extensive wound management resources but also mitigates indirect costs associated with lost productivity and prolonged disability. Moreover, through preventing complications such as infection or chronic pain that often accompany traditional treatments, this approach could further reduce the likelihood of long-term healthcare expenditures.

Enhancing the regenerative capabilities of wound care through such innovative therapies could substantially alleviate the public health burden of treating complex injuries. It could enable quicker patient recovery and a faster return to work, which are critical components in improving quality of life and reducing the economic impact on both individuals and healthcare systems. Therefore, advancing this technique through rigorous research could have profound implications for public health policy and clinical practices worldwide.

## 4. Limitations

While this study offers valuable insights into the potential benefits of autologous fat grafting combined with platelet-rich plasma and growth factors, there are several inherent limitations due to its clinical case design. The surgical approach used—autologous fat grafting—was an alternative to the standard treatment (local or free microsurgical skin flap), chosen because the patient declined the standard treatment. Additionally, the current literature on the use of autologous fat grafting as an alternative to flaps for tendon exposure coverage in the upper extremity is limited, which restricts the scope of discussion in this report and underscores its significance. It is important to note that platelet counting was not performed on the platelet-rich plasma preparations in accordance with established protocols, though incorporating this step could enhance the accuracy of the procedure’s replication and potentially improve the success rate of similar cases. Lastly, the plastic surgery team did not employ negative pressure therapy, given their limited experience with autologous fat grafting as an alternative to flaps for tendon coverage in the upper extremity. Despite the possibility of achieving similar results in a shorter time via combining fat grafting with negative pressure, this report remains valuable as it highlights a successful outcome in a constrained setting.

## 5. Conclusions

Autologous fat tissue graft combined with PRP and growth factors has proven to be a highly effective alternative to traditional flap procedures for covering exposed tendons. This method not only facilitates skin regeneration but also positively influences the behavior of dermal and epidermal cells through the bioactive compounds in its “secretome”. The technique’s relative ease of access, cost-effectiveness, nonimmunogenic nature, and potential to enhance aesthetic outcomes make it an attractive option in clinical scenarios involving tissue exposure. Its application could revolutionize the approach to wound healing in plastic and reconstructive surgery, providing a simpler, quicker, and potentially less costly alternative to more invasive methods.

## Data Availability

Due to the nature of this case report, the data supporting the findings cannot be shared publicly in order to protect patient confidentiality. However, further details may be made available by the corresponding author upon reasonable request.

## Supplementary Materials

The following supporting information can be downloaded at: https://www.mdpi.com/article/10.3390/jcm13185640/s1, Supplementary Material S1: Video S1: Full Functional Recovery: Demonstrating Complete Flexion and Extension in Fingers 2–5. Supplementary Material S2: Patient responses to DASH questionnaire.
