# Supplementary material for: Flap-Free Tendon Coverage Using Autologous Fat Grafts Enhanced with Platelet-Rich Plasma and Growth Factors at a Secondary Level Hospital: A Case Report
Source: J Clin Med. 2024 Sep 23;13(18):5640. doi: 10.3390/jcm13185640 (PMC11432800; doi:10.3390/jcm13185640)
Supplement: Supplementary file 1 [file jcm-13-05640-s001.zip › Supplementary Material S2.pdf]

Supplementary File S2. Patient responses to DASH questionnaire.

INSTRUCTIONS:

This questionnaire asks you about your symptoms as well as your ability to perform certain activities or tasks. Please answer each question based on your condition or ability during the past week. To do this, circle the appropriate number. If you did not have the opportunity to do any of the activities during the last week, please try to approximate the answer that you consider to be the most accurate. It doesn't matter which hand or arm you use to perform the activity; Please answer based on your ability or ability and how you can carry out said task or activity.

Please rate your ability or ability to do the following activities during the last week. To do this, circle the appropriate number for each answer.

1. Open a new glass jar.

1. No difficulty

2. Little difficulty

3. Moderate difficulty

4. A lot of difficulty

5. Incapable

2. Write.

1. No difficulty

2. Little difficulty

3. Moderate difficulty

4. A lot of difficulty

5. Incapable

3. Prepare the food.

1. No difficulty

2. Little difficulty

3. Moderate difficulty

4. A lot of difficulty

5. Incapable

4. Turn the key to open the door or start the car.

1. No difficulty

2. Little difficulty

3. Moderate difficulty

4. A lot of difficulty

5. Incapable

5. Push and open a heavy door

1. No difficulty

2. Little difficulty

3. Moderate difficulty

4. A lot of difficulty

5. Incapable

6. Place an object on shelves above your head

1. No difficulty

2. Little difficulty

3. Moderate difficulty

4. A lot of difficulty

5. Incapable

7. Perform hard housework such as mopping the floor, cleaning walls and glass, etc.

1. No difficulty

2. Little difficulty

3. Moderate difficulty

4. A lot of difficulty

5. Incapable

8. Fix the garden or do work in the field.

1. No difficulty

2. Little difficulty

3. Moderate difficulty

4. A lot of difficulty

5. Incapable

9. Make the beds.

1. No difficulty

2. Little difficulty

3. Moderate difficulty

4. A lot of difficulty

5. Incapable

10. Carry a grocery bag or carry a briefcase.

1. No difficulty

2. Little difficulty

3. Moderate difficulty

4. A lot of difficulty

5. Incapable

11. Carrying a heavy object (more than 5 kg)

1. No difficulty

2. Little difficulty

3. Moderate difficulty

4. A lot of difficulty

5. Incapable

12. Change a light bulb in the ceiling.

1. No difficulty

2. Little difficulty

3. Moderate difficulty

4. A lot of difficulty

5. Incapable

13. Wash and dry your hair.

1. No difficulty

2. Little difficulty

3. Moderate difficulty

4. A lot of difficulty

5. Incapable

14. Wash your back.

1. No difficulty

2. Little difficulty

3. Moderate difficulty

4. A lot of difficulty

5. Incapable

15. Put on a sweater or sweater.

1. No difficulty

2. Little difficulty

3. Moderate difficulty

4. A lot of difficulty

5. Incapable

16. Use a knife to cut food.

1. No difficulty

2. Little difficulty

3. Moderate difficulty

4. A lot of difficulty

5. Incapable

17. Entertainment activities that require little effort such as knitting, sewing, playing cards or dominoes.

1. No difficulty

2. Little difficulty

3. Moderate difficulty

4. A lot of difficulty

5. Incapable

18. Activities that require some effort on your arm, shoulder, or hand, such as using a hammer, playing golf, tennis, or bocce.

1. No difficulty

2. Little difficulty

3. Moderate difficulty

4. A lot of difficulty

5. Incapable

19. Activities that require your arm to move freely such as swimming

1. No difficulty

2. Little difficulty

3. Moderate difficulty

4. A lot of difficulty

5. Incapable

20. Driving.

1. No difficulty

2. Little difficulty

3. Moderate difficulty

4. A lot of difficulty

5. Incapable

21. Sexual activity.

1. No difficulty

2. Little difficulty

3. Moderate difficulty

4. A lot of difficulty

5. Incapable

22. During the past week, have your shoulder, arm, or hand problems interfered with your regular social activity with family, friends, or coworkers?

1. No. Not at all

2. Little

3. Regular

4. Quite limited

5. Impossible to do

23. During the last week have you had difficulty doing your work or other daily activities due to problems with your shoulder, arm, or hand?

1. No. Not at all

2. Little

3. Regular

4. Quite limited

5. Impossible to do

Please rate the severity or severity of the following symptoms.

24. Pain in the shoulder, arm or hand.

1. None

2. Mild

3. Moderate

4. Serious

5. Very serious

25. Shoulder, arm or hand pain when doing any specific activity:

1. None

2. Mild

3. Moderate

4. Serious

5. Very serious

26. Feeling of cramps, tingling or electricity in the shoulder, arm or hand.

1. None

2. Mild

3. Moderate

4. Serious

5. Very serious

27. Weakness or lack of strength in the shoulder, arm or hand.

1. None

2. Mild

3. Moderate

4. Serious

5. Very serious

28. Stiffness or lack of mobility in the shoulder, arm or hand.

1. None

2. Mild

3. Moderate

4. Serious

5. Very serious

29. During the past week, have you had difficulty sleeping due to shoulder, arm, or hand pain?

1. None

2. Mild

3. Moderate

4. Serious

5. Very serious

30. "I feel less capable, confident, and useful because of my problem with my shoulder, arm, or hand.

1. Completely false

2. False

3. I don't know

4. True

5. Completely true

WORK MODULE (Optional)

The following questions ask about the impact of your arm, shoulder or hand problem.

on your ability to work (including household chores if that is your main job)

Please indicate your job/occupation: **University teacher**

I don't work (you can skip this section).

Circle the number that best describes your physical ability in the past week:

Did you have any difficulty using your usual work technique?

1. No difficulty
2. Little difficulty
3. Moderate difficulty
4. A lot of difficulty
5. Impossible

Did you have any difficulty doing your usual work because of shoulder, arm, or hand pain?

1. No difficulty
2. Little difficulty
3. Moderate difficulty
4. A lot of difficulty
5. Impossible

Did you have any difficulty doing your job as well as you would like?

1. No difficulty
2. Little difficulty
3. Moderate difficulty
4. A lot of difficulty
5. Impossible

Did you have any difficulty spending the usual amount of time on your job?

1. No difficulty
2. Little difficulty
3. Moderate difficulty
4. A lot of difficulty
5. Impossible
